# Molecular detection and phylogenetic identification of *Toxoplasma gondii*-like strains, *Hepatozoon ixoxo* and *Lankesterella* sp. in frogs and toads

**DOI:** 10.3389/fvets.2025.1568298

**Published:** 2025-03-31

**Authors:** Mubashra Salim, Asmat Ullah Khan, Alaudin Khan Niazi, Amna Aqdas, Turki M. Dawoud, Muhammad Usman, Hira Muqaddas, Shakir Ullah, Hanène Belkahia, Adil Khan, Mourad Ben Said, Furhan Iqbal

**Affiliations:** ^1^Institute of Zoology, Bahauddin Zakariya University, Multan, Pakistan; ^2^Department of Basic and Applied Zoology, Shaheed Benazir Bhutto University, Sheringal, Dir Upper, Khyber Pakhtunkhwa, Pakistan; ^3^Department of Chemistry and Biochemistry, Texas Tech University, Lubbock, TX, United States; ^4^Department of Botany and Microbiology, College of Science, King Saud University, Riyadh, Saudi Arabia; ^5^The University of Lahore, Lahore, Pakistan; ^6^Department of Zoology, The Women University Multan, Multan, Pakistan; ^7^Department of Zoology, Abdul Wali Khan University Mardan, Multan, Pakistan; ^8^Laboratory of Microbiology, National School of Veterinary Medicine of Sidi Thabet, University of Manouba, Manouba, Tunisia; ^9^Department of Zoology, Bacha Khan University, Charsadda, Pakistan; ^10^Department of Basic Sciences, Higher Institute of Biotechnology of Sidi Thabet, University of Manouba, Manouba, Tunisia

**Keywords:** *Bufo olivaceous*, *Bufo stomaticus*, *Hoplobatrachus tigerinus*, prevalence and phylogeny, amphibians, Pakistan

## Abstract

**Introduction:**

Despite Pakistan’s rich amphibian fauna, frog and toad species remain largely unexplored regarding blood-borne parasites.

**Methods:**

This study aims to investigate potential infections by *toxoplasma gondii* and *Hepatozoon* spp. in four amphibian species (*n* = 223) collected from various regions in Punjab and Khyber Pakhtunkhwa provinces.

**Results:**

Molecular analyses revealed that 17 out of 223 amphibians (7.6%) were infected with *Hepatozoon* spp., with the highest infection rates found in *Bufo olivaceous* (20.0%), followed by *Bufo stomaticus* (9.3%) and *Hoplobatrachus tigerinus* (5.05%). DNA sequencing and BLAST analysis confirmed the presence of *Hepatozoon ixoxo* and *Lankesterella* sp. phylogenetic analysis of both pathogens demonstrated genetic diversity among the Pakistani isolates, clustering with isolates from birds, amphibians, and reptiles worldwide. To the best of our knowledge, this is first ever report globally where we are documenting that 4.5% of the screened Pakistani anurans, including frogs (*H. tigerinus*, 8.1%) and toads (*B. stomaticus*, 1.9%), were infected with *toxoplasma gondii*-like strains. Parasite prevalence varied between sampling sites and amphibian species. This study represents the first report from Pakistan documenting the prevalence and genetic characterization of *Hepatozoon* sp., *Lankesterella* sp., and *T. Gondii*-like strains among amphibians. We recommend conducting similar large-scale studies across various geo-climatic regions of Pakistan to further explore the epidemiology, genetic diversity, host–parasite interactions, and effective control of these pathogens among local frog and toad species. Identifying genetically related *T. Gondii* strains in unexpected host animals, such as amphibians, has been crucial for contributing to the elucidation of the parasite’s evolutionary history.

## 1 Introduction

Amphibians, a diverse group of vertebrates, require water or moist environments for survival (1). Globally, there are currently 7,947 amphibian species, with 7,013 species being frogs and toads (Anura) found in various aquatic and terrestrial habitats, excluding estuarine and marine environments (2). Anurans are notable for their feeding habits, medicinal value, and their economic, esthetic and cultural significance (3). They are also consumed as food internationally (4).

In Pakistan, information on anurans distribution is limited due to the lack of attention from the scientific community (5). The country’s amphibian fauna is solely represented by the order Anura, comprising 21 species, 12 genera, and 4 families: Ranidae, Microhylidae, Bufonidae, and Megophryidae (3). Amphibians in Pakistan are reported from the Indus valley in the west bank of the river, runnel of Himalayan North region, water channel and sub-mountainous regions of western Baluchistan (6). Despite their diversity, anurans are experiencing the highest rate of population decline among vertebrates (7). Factors contributing to this decline include anthropogenic activities such as deforestation, industrialization, urbanization, mechanized agriculture, pesticide use, road causalities and vector borne diseases (3).

Parasites can negatively affect their hosts, leading to disturbed physiology and decreased reproductive success (8). *Hepatozoon* species, belonging to the family Hepatozoidae, are apicomplexan protozoans that target the red and white blood cells of various hosts, including reptiles, amphibians, and mammals (9, 10). These species have also been reported in several invertebrate species that primarily act as vectors, transmitting the parasites from invertebrates to vertebrates and among different vertebrate species (11, 12). A common mode of transmission for *Hepatozoon* spp. is through the ingestion of an infected invertebrate or intermediate prey, leading to the release of developmental stages in endothelial cells, hepatocytes, and other visceral organs of a wide variety of vertebrate hosts (13). *Toxoplasma gondii* is an intracellular protozoan that infects nearly all warm-blooded animals, both domestic and wild (14, 15). Its prevalence in wildlife is closely linked to the presence of felids, the definitive hosts, as the parasite’s oocysts are excreted in feces and subsequently ingested by new hosts (16, 17). Common symptoms of toxoplasmosis in animals include fever, loss of appetite, and lethargy, which can vary depending on whether the infection is acute or chronic (18).

In Pakistan, there are limited reports documenting amphibian diversity, and information regarding the endo-parasites infecting frogs and toads is scarce (3, 19, 20). To fill this knowledge gap, blood samples were collected from anuran species across six districts in Punjab and Khyber Pakhtunkhwa provinces and screened for the DNA of Haemogregarines and *T. gondii* and related strains complex using PCR and sequencing methods. Additionally, risk factors associated with infection were evaluated. The findings offer valuable insights into the epidemiology of these parasites in Pakistan, with significant implications for wildlife conservation and public health.

## 2 Materials and methods

### 2.1 Study areas and subjects

**Figure 1:**
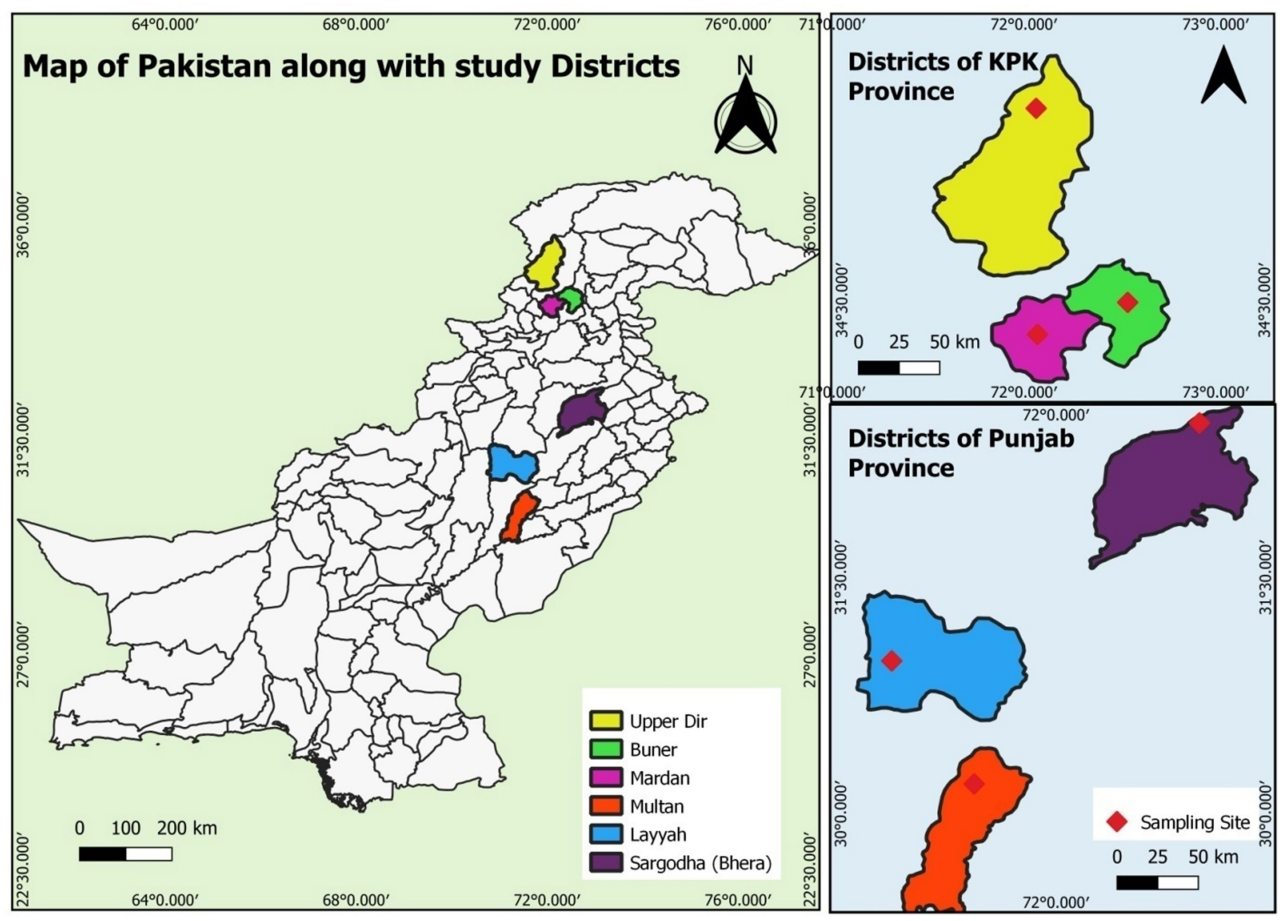
Magnified map of Punjab and KPK showing different sampling sites (Multan, Layyah, Sargodha, Upper Dir, Buner, and Mardan).

Samples were collected from three regions in Punjab [Layyah, Multan and Sargodha (Bhera)] and three regions in Khyber Pakhtunkhwa (KPK) [Upper Dir (Sheringal), Mardan and Buner] in Pakistan. The survey took place during the summer and rainy months (July to October) over two consecutive years (2022–2023). The study areas in the two provinces were geographically and climatically distinct, allowing for the observation of geo-climatic differences on the prevalence of studied parasites (Figure 1).

### 2.2 Sample collection and identification

A total of 223 amphibians, representing four different species of toads and frogs, were collected from three regions each in Punjab (*n* = 126) and KPK (*n* = 97). Samples were collected during the summer and rainy months as during autumn and winters these animals hibernate being cold blooded animals. Frogs and toads were caught by hand or using nets and transported in clean, ventilated plastic containers to the Molecular Parasitology Laboratory at the Institute of Zoology, Bahauddin Zakariya University, Multan. The specimens were identified following the methodologies of Ingerm and Stuebing (21) and Frost *et al*. (1). Briefly, frogs can be differentiated as they have long legs, longer than their head and body, used for hopping. Toads, on the other hand, have much shorter legs and prefer to crawl. Frogs have smooth, somewhat slimy skin while toads have dry, warty skin (6).

### 2.3 Data and blood collection

A questionnaire was completed for each animal to gather information about each animal in order to calculate the prevalence of studied parasites according to each risk factor. The captured frogs and toads were anesthetized and blood samples (> 0.1 mL) were collected from each animal via cardiac puncture with a disposable syringe. The blood was transferred into screw capped tubes containing EDTA for DNA extraction.

### 2.4 DNA extraction from blood

Genomic DNA was extracted from the blood of each frog and toad using the blood genomic DNA extraction kit (Solarbio, China) following the manufacturer’s instructions.

### 2.5 Parasite molecular detection by PCR

The extracted DNA samples were analyzed for the presence of *Hepatozoon* spp., by targeting their 18S rRNA gene, using the forward primer HepF300 5′-GTT TCT GAC CTA TCA GCT TTC GAC G-3′ and reverse primer Hep900 5′-C AAA TCT AAG AAT TTC ACC TCT GAC-3′, following the PCR protocol reported by Ujvari *et al*. (22). The primers amplified a 600 base pair fragment from 18S rRNA gene that is shared by variety of species in addition to *Hepatozoon* spp. (23). For the detection of *T. gondii* and strains genetically related, the *ITS-1* gene was targeted using only the internal primers of the nested PCR protocol (ITS_fw 5′-GAT TTG CAT TCA AGA AGC GTG ATA GTA T − 3′ and ITS_rv 5′-AGT TTA GGA AGC AAT CTG AAA GCA CAT C -3′) as reported by Zintl et al. (24).

DNA amplification was carried out in a DNA thermal cycler (Gene Amp® PCR system 2,700 Applied Biosystems Inc., United Kingdom). During each reaction, distilled water was used as a negative control, while DNA from positive animals (for screened parasites available at our laboratory from previous studies) was used as a positive control.

### 2.6 DNA sequencing and phylogenetic analysis

Amplified PCR products of all parasites were sequenced by a commercial company (First base, Malaysia), and the resultant partial gene sequences were submitted to NCBI GenBank. The newly generated sequences targeting the 18S SSU gene (600 bp) were compared with other isolates from *Lankesterella* and *Hepatozoon* spp. available on GenBank. Similarly, the *ITS-1* partial sequences (300 bp) of *T. gondii*-like strains were compared with those isolated from *T. gondii* species previously deposited in GenBank. The sequences were aligned using Geneious version 7.1.3 (25) with the MUSCLE algorithm^1^ and default settings, including related sequences identified through a Blastn search.

For phylogenetic analyses, Bayesian inference (BI) and Maximum Likelihood (ML) methods were performed. The program JModeltest v.2.1.10 (26) was used for the ML method to identify the best evolutionary model. Based on the Akaike Information Criterion (AIC), the transitional model with a discrete Gamma distribution (TVM + G) was chosen (55). The analysis was inferred using PhyML (27) with 1,000 bootstrap replicates (>50%). For BI analysis, MrBayes was implemented using the computational resource CIPRES (28). The best BIC score indicated the general time reversible model (GTR + I + *Γ*) (29). In addition, the Markov chain Monte Carlo (MCMC) algorithm was run for 10,000,000 generations, sampling one tree every 1,000 generations. For burn-in, the first 25% of generations were discarded, and the consensus tree was estimated using the remaining trees. Bayesian posterior probabilities (BPP) cut-off considered was >50%. The BI and ML constructed trees were edited in FigTree v1.4 (30). *Adelina dimidiata* Schneider, 1875, *Adelina grylli* (DQ096835 and DQ096836) and *Klossia helicina* Schneider, 1875 (HQ224955) from the suborder Adeleorina were used as outgroups during the phylogenetic analysis of *Hepatozoon. Dactylosoma piperis* (MW264134), *Dactylosoma ranarum* (HQ224958), and *Dactylosoma kermiti* (MN839798) (Apicomplexa, Dactylosomatidae) were used as outgroups for the *Lankesterella* spp. genetic diversity analysis. While *Hammondia* (AF159240 and KJ394594) and *Sarcocystis* species (KF601312, KM657771, EF079887, MG493471, AY082645, AY082647 and AF098245) were used as outgroups for *T. gondii* and related strains’ analysis.

### 2.7 Statistical analysis

The statistical analysis of data was performed using Minitab (Minitab, Pennsylvania, United States). A *p*-value of less than 0.05 was considered statistically significant. The PCR-based pathogen prevalence between various sampling sites and anuran species was compared using one way ANOVA. The association between the presence of each pathogen and the studied epidemiological factors were assessed using contingency table analysis with Fisher’s exact test (for 2 × 2 tables).

## 3 Results

### 3.1 Taxonomic identification of captured anurans

The captured amphibians included one frog species [*Hoplobatrachus tigerinus* (*N* = 99)] and three toad species [*Bufo stomaticus n* = 108), *Bufo olivaceous* (*n* = 10) and *Bufo melanostictus n* = 6)].

### 3.2 Prevalence of *Hepatozoon* spp. among captured anurans

A total of four anuran species were captured and identified during this investigation including one frog (*H. tigerinus*) and three toad (*B. stomaticus, B. olivaceous*, and *B. melanostictus*) species. Polymerase chain reaction amplified a 600 base pairs fragment from the 18S rRNA gene of *Hepatozoon* spp. in 17 out of 223 (7.6%) frog and toad blood samples collected from two provinces in Pakistan (Table 1).

**Table 1:** Haemogregarines prevalence among frog and toad species in overall and according to the six sampling districts.

Provinces	Districts	Frogs				Toads				*p* value^3^
		*Hoplobatrachus tigerinus*		*Bufo stomaticus*		*Bufo olivaceous*		*Bufo melanostictus*		
		Positive/Total (% ± C.I.^1^)	*p* value^2^	Positive/Total (% ± C.I.^1^)	*p* value^2^	Positive/Total (% ± C.I.^1^)	*p* value^2^	Positive/Total (% ± C.I.^1^)	*p* value^2^	
Punjab	Layyah	3/27 (11 ± 0.117)	0.271	2/17 (12 ± 0.152)	0.721	2/8 (25 ± 0.299)	0.453	–	#	0.581
	Multan	2/24 (8 ± 0.109)		0/3 (0)		0/2 (0)		–		0.799
	Sargodha	0/20 (0)		2/19 (11 ± 0.137)		–		0/6 (0)		0.238
KPK	Upper Dir	–		6/55 (11 ± 0.082)		–		–		#
	Buner	0/10 (0)		0/14 (0)		–		–		#
	Mardan	0/18 (0)		-		–		–		#
Total		5/99 (5.05 ± 0.043)		10/108 (9.3 ± 0.054)		2/10 (20 ± 0.246)		0/6 (0)		0.260

*p > 0.05 = Non significant. 1C.I.: 95% confidence interval, 2p value calculated between districts for each amphibian species, 3p value calculated between amphibian species for each district, −Absence of sampling in this district, #Statistical analysis was not possible.*

### 3.3 Genetic analysis and phylogenetic positioning of *Hepatozoon* and *Lankesterella* spp

**Figure 2:**
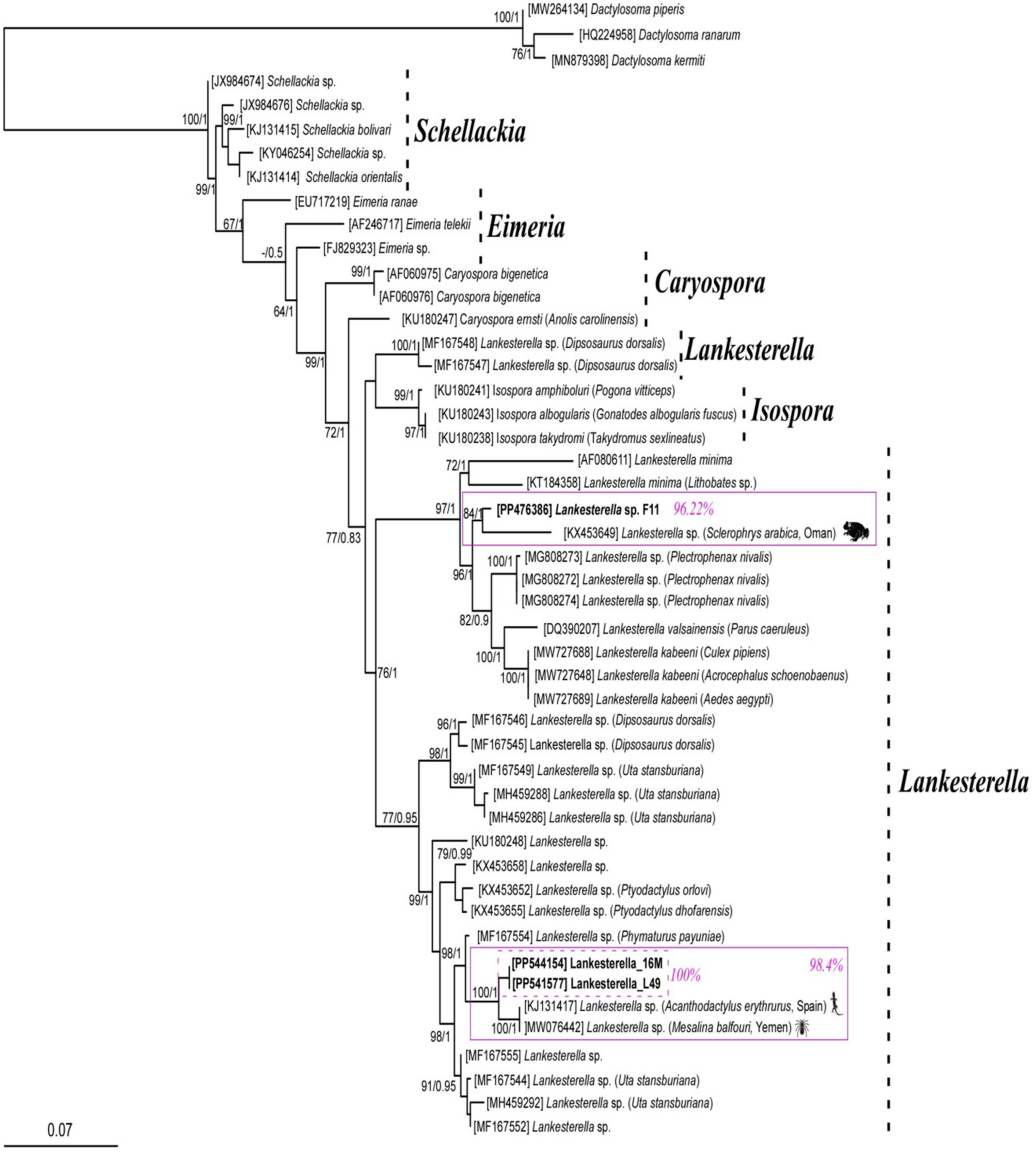
Phylogenetic tree of *Hepatozoon* spp. based on the partial 18S rRNA gene sequences. New sequence of *Hepatozoon* sp. obtained in this study (PP481405) is highlighted in bold. Scale bar represents 0.2 substitutions per nucleotide positions. Bootstrap values are shown as numbers on the nodes.

**Figure 3:**
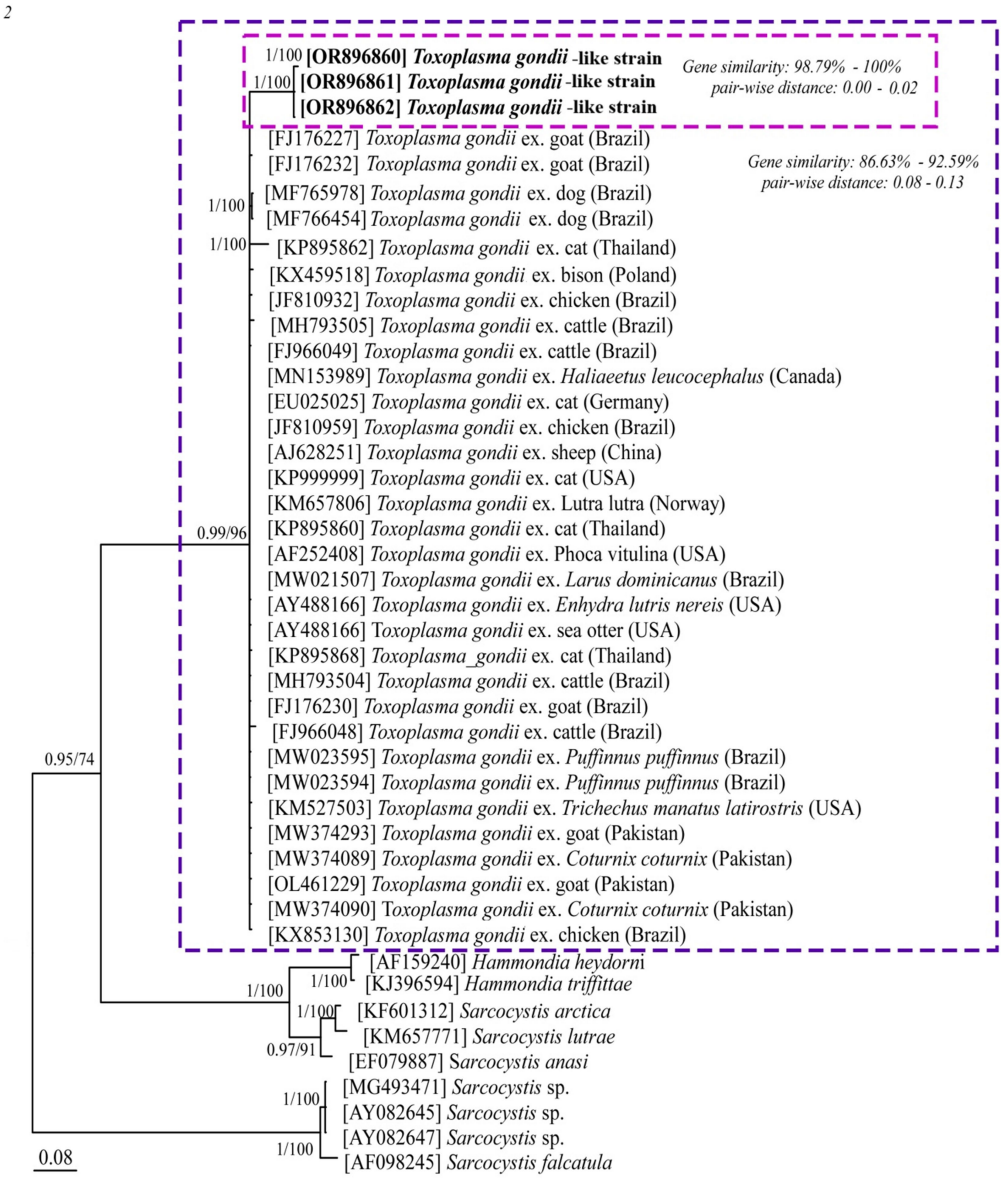
Phylogenetic tree of *Lankesterella* sp. based on the partial 18S rRNA gene sequences. The three new sequences of *Lankesterella* sp. obtained in this study (PP476386, PP544154 and PP541577) are highlighted in bold and presented in box. Scale bar represents 0.07 substitutions per nucleotide position. Bootstrap values are shown as numbers on the nodes.

BLAST analysis of the amplified parasite sequences confirmed that our Pakistani anurans were infected with both *Hepatozoon* spp. and *Lankesterella* spp., as anticipated based on the generalized primers capable of amplifying the 18S rRNA gene from various organisms. The analysis revealed that our *Hepatozoon* sp. sequence (PP481405) is genetically closest to *Hepatozoon ixoxo*, showing 99.77% identity with an isolated sequence (KP119772). Similarly, our Lankesterella sp. sequence (F11, PP476386) is genetically similar to *Lankesterella* sp. (KX453649) with 96.22% identity. To further explore genetic diversity, we analyzed *Hepatozoon* spp. and *Lankesterella* spp. separately. The haplotype PP481405 for *Hepatozoon* spp. clustered with sequences from amphibians and reptiles in South Africa (MG519501, MG519504, KP119772, KP119773, MG041591, MG041598, and MG041600), Canada (JN181157, HQ224960, and HQ224962), Brazil (MW591556), and the United States (AF176837) (Figure 2). For *Lankesterella* spp., the two haplotypes identified (PP544154 and PP541577) were genetically similar and formed a monophyletic cluster with 100% bootstrap support. Our third haplotype (PP476386) was clustered separately. These haplotypes also displayed similarities with global *Lankesterella* spp. sequences from birds, amphibians, and reptiles (Figure 3). Notably, Pakistani haplotypes PP544154 and PP541577 clustered with *Lankesterella* sp. from Spain (KJ131417) and Yemen (MW076442), while isolate PP476386 clustered with *Lankesterella* sp. from Oman (KX453649) (Figure 3).

For *Lankesterella* spp., the two haplotypes revealed in this study (PP544154 and PP541577) were genetically similar and clustered together with a monophyletic branch with same number of substitutions at 100% bootstrap support values. Our third haplotype (PP476386) was clustered separately from the other two haplotypes. These haplotypes also showed similarities with *Lankesterella* spp. sequences reported from birds, amphibian, and reptiles globally (Figure 3). Pakistani haplotypes PP544154 and PP541577 clustered with *Lankesterella* sp. reported from Spain (KJ131417) and Yemen (MW076442). The remaining Pakistani isolate PP476386 clustered with *Lankesterella* sp. from Oman (KX453649) (Figure 3).

### 3.4 Risk factor analysis for *Hepatozoon* spp

When comparing the prevalence of *Hepatozoon* spp. among the anurans (frogs and toads) captured from various sampling sites, one-way ANOVA results indicated that prevalence of this parasite was not restricted to a particular sampling site (*p* = 0.260) (Table 1). Fisher’s exact test showed that *Hepatozoon* spp. infection was not associated with the sex of *H. tigerinus* captured from six districts in Pakistan (*p* = 0.631) (Table 2). A similar trend was observed for the three toad species included in this study, with Fisher’s exact test indicating that *Hepatozoon* spp. infection was not related to the sex of *B. stomaticus* (*p* = 0.589) and *B. olivaceous* (*p* = 0.617). This analysis was not possible for *B. melanostictus* as none of the toad belonging to this species was found infected with *Hepatozoon* spp. (Table 2).

**Table 2:** Prevalence rates of haemogregarines in amphibian species by sex.

Amphibian species	Sex classes	Positive/Total (% ± C.I.^1^)	*p* value^2^
*Hoplobatrachus tigerinus*	Male	2/50 (4 ± 0.054)	0.631
	Female	3/49 (6 ± 0.066)	
*Bufo stomaticus*	Male	4/52 (8 ± 0.072)	0.589
	Female	6/56 (11 ± 0.080)	
*Bufo olivaceous*	Male	2/9 (22 ± 0.272)	0.617
	Female	0/1 (0)	
*Bufo melanostictus*	Male	0/5 (0)	#
	Female	0/1 (0)	

*p > 0.05 = Non significant. 1C.I.: 95% confidence interval, 2p value calculated between sexes for each amphibian species, −Absence of sampling in this district, #Statistical analysis was not possible.*

### 3.5 Prevalence of *Toxoplasma gondii*-like strains among captured anurans

Polymerase chain reaction amplified a 300 base pair fragment specific to the *ITS-1* marker of *T. gondii*-like strains in 10 out of 223 (4.5%) frog and toad blood samples collected from three sampling districts (Layyah, Sargodha and Multan) in Punjab and three sampling districts (Upper Dir, Buner and Mardan) in KPK during present study (Table 3).

**Table 3:** *Toxoplasma gondii*-like strains’ prevalence rates among different species of frogs and toads in overall and according to the six sampling districts.

Provinces	Districts	Frogs				Toads				*p* value^3^
		*Hoplobatrachus tigerinus*		*Bufo stomaticus*		*Bufo olivaceous*		*Bufo melanostictus*		
		Positive/Total (% ± C.I.^1^)	*p* value^2^	Positive/Total (% ± C.I.^1^)	*p* value^2^	Positive/Total (% ± C.I.^1^)	*p* value^2^	Positive/Total (% ± C.I.^1^)	*p* value^2^	
Punjab	Layyah	3/27 (11 ± 0.117)	0.043^*^	2/17 (12 ± 0.152)	0.027^*^	0/8 (0)	#	–	#	0.593
	Multan	5/24 (21 ± 0.162)		0/3 (0)		0/2 (0)		–		0.532
	Sargodha	0/20 (0)		0/19 (0)		-		0/6 (0)		#
KPK	Upper Dir	–		0/55 (0)		-		–		#
	Buner	0/10 (0)		0/14 (0)		-		–		#
	Mardan	0/18 (0)		-		-		–		#
Total		5/99 (5.05 ± 0.043)		2/108 (1.9 ± 0.025)		0/10 (0)		0/6 (0)		0.0513

^1^C.I.: 95% confidence interval, ^2^*p* value calculated between districts for each amphibian species, ^3^*p* value calculated between amphibian species for each district, ^*^Statistically significant, *p* < 0.05.

### 3.6 BLAST analysis and phylogenetic study of *ITS-1* region of *Toxoplasma gondii*-like strains

**Figure 4:**
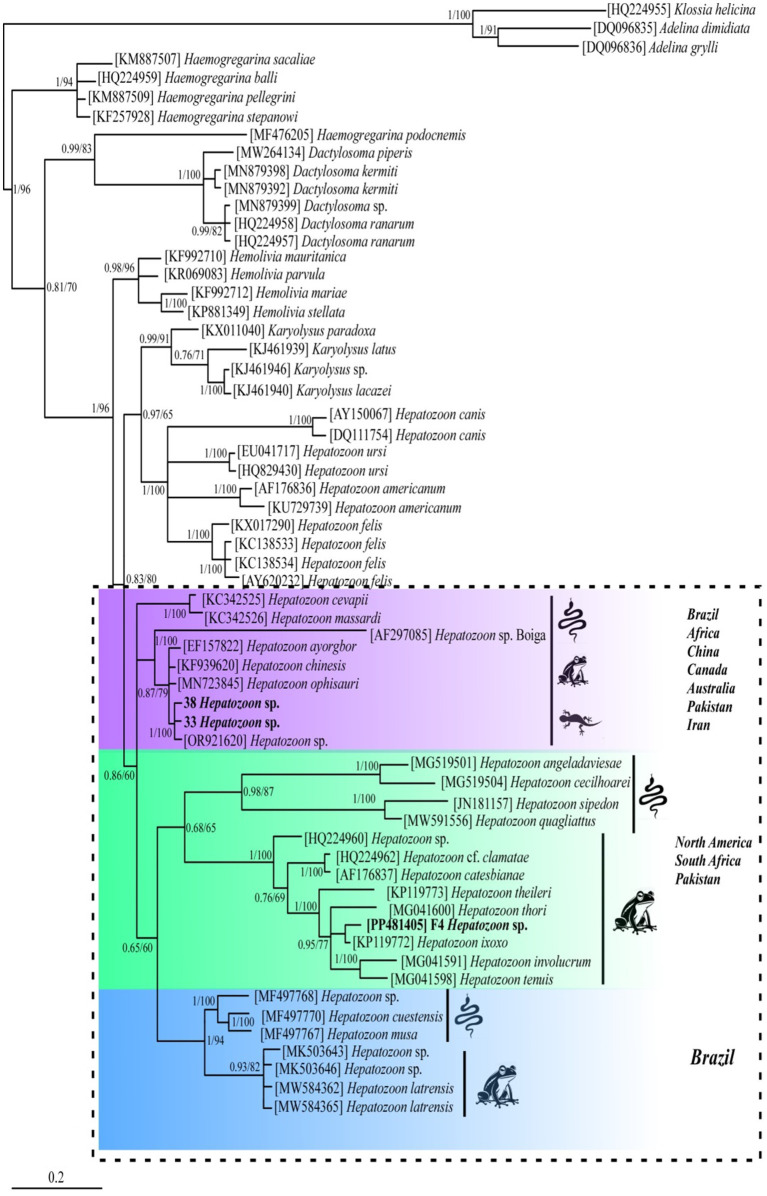
Phylogenetic tree of *Toxoplasma gondii* and related strains based on ITS-1 partial sequences. The three new sequences of *T. gondii-*like strains obtained in this study (OR896860, OR896861, and OR896862) are highlighted in bold and presented in box. Scale bar represents 0.08 substitutions per nucleotide position. Bootstrap values are shown as numbers on the nodes.

BLAST analysis conducted on haplotypes of our *T. gondii*-like strains revealed a sequence identity of 90.57% with *T. gondii* isolates reported from various mammals and birds across different countries (FJ176227, MH793500, KP895860, and MW374089). Sequence comparisons among our haplotypes showed a high identity of 98.87%. Phylogenetic analysis of the *ITS-1* region of *T. gondii* and related strains indicated that the three Pakistani haplotypes generated in this study (OR896860, OR896861, and OR896862) clustered together but were genetically distinct from the *ITS-1* sequences of *T. gondii* reported in various mammals and birds in Brazil (FJ176227, FJ176230, FJ176232, FJ966048, MF765978, MF766454, JF810932, JF810959, MH793504, MH793505, FJ966049, MW021507, MW023594, MW023595, and KX853130), as well as in Thailand (KP895860, KP895862, and KP895868), Poland (KX459518), Canada (MN153989), Germany (EU025025), China (AJ628251), the United States (KP999999, AF252408, AY488166, and KM525503), Norway (KM657806), and Pakistan (MW374089, MW374090, MW374293, and OL461229) (Figure 4).

### 3.7 Risk factor analysis for *Toxoplasma gondii*-like strains

When comparing the prevalence of *T. gondii*-like strains among anurans (frogs and toads) captured from various sampling sites, One-way ANOVA results indicated a significant variation in parasite prevalence between sampling sites. The infected anurans included one frog species (*H. tigerinus*; *p* = 0.043) and one toad species (*B. stomaticus*; *p* = 0.027), all of which were captured from Punjab province. No infections were found in animals captured from KPK during present investigation (Table 3). Fisher’s exact test analysis revealed that *T. gondii*-like strains’ infection was not associated with the sex of *H. tigerinus* captured from five districts in Pakistan (*p* = 0.481; Table 4). A similar trend was observed for *B. stomaticus* (*p* = 0.958). This analysis could not be performed for *B. olivaceous* and *B. melanostictus* as no infections were detected in these toad species (Table 4).

**Table 4:** Prevalence rates of *Toxoplasma gondii-*like strains in amphibian species by sex.

Amphibian species	Sex classes	Positive/Total (% ± C.I.^1^)	*p* value^2^
*Hoplobatrachus tigerinus*	Male	5/50 (10 ± 0.082)	0.481
	Female	3/49 (6 ± 0.066)	
*Bufo stomaticus*	Male	1/52 (8 ± 0.037)	0.958
	Female	1/56 (2 ± 0.035)	
*Bufo olivaceous*	Male	0/9 (0)	#
	Female	0/1 (0)	
*Bufo melanostictus*	Male	0/5 (0)	#
	Female	0/1 (0)	

*p > 0.05 = Non significant. 1C.I.: 95% confidence interval, 2p value calculated between sexes for each amphibian species, −Absence of sampling in this district, #Statistical analysis was not possible.*

## 4 Discussion

Amphibians are a transitional group of tetrapods that remain closely tied to water, particularly for reproduction, and they are among the most threatened vertebrate groups in the world. The main causes include climate change, habitat destruction and emerging diseases (23). As ectotherms with permeable skin, amphibians are extremely vulnerable to habitat alterations as well as for parasitic infections (31). They are known to host a wide variety of hemoparasites, including *Haemogregarina*, *Hepatozoon*, *Lankesterella* and *Schellackia* (32, 33). The presence of *Toxoplasma gondii* has never been reported in cold blooded animals before, especially in anurans. In light of these facts, the present study was designed to report the molecular prevalence and phylogeny of *Hepatozoon* spp. and *T. gondii*-like strains among various frog and toad species captured from three districts each in Punjab and KPK (Pakistan).

In the past decade, the understanding of apicomplexan parasite diversity in amphibians has significantly advanced due to the use of molecular techniques (34, 35). Despite this progress, the molecular prevalence of *Hepatozoon* spp. has not been previously reported in any animal species in Pakistan, particularly in anurans. Our study provides the first data on this unexplored research area in Pakistan. We found that 7.6% of anurans from the family Ranidae and Bufonidae were infected with *Hepatozoon* spp., including one frog species (*H. tigerinus*) and three toad species (*B. stomaticus, B. olivaceous* and *B. melanostictus*) (Table 1). DNA sequence analysis indicated that our primers amplified two parasite species: *Hepatozoon* spp. and *Lankesterella* sp. consistent with previous findings that these primers can detect multiple species (23).

Few reports exist on the presence of *Hepatozoon* spp. and/or *Lankesterella* sp. in anurans worldwide. Our findings are in line with those of Úngari et al. (36), who reported a 6.06% prevalence of *Hepatozoon* spp. in anurans from Mato Grosso State, Brazil. In another Brazilian study, de Abreu Reis Ferreira et al. (23) reported a 100% prevalence rate of haemogregarines parasites in *Leptodactylus latrans* and the first molecular report of *Lankesterella* sp. in amphibians from Brazil. Similarly, Isaak Delgado et al. (37) reported an 85% infection rate of *Hepatozoon* sp. and a 57% prevalence of *Lankesterella* sp. in *Lithobates vaillanti* from La Florida Biological Station, Tabasco, Mexico. Netherlands et al. (32) also reported a 73% infection rate of *Hepatozoon* spp. among African anurans of the family Bufonidae in Northern, Southern and Central Africa. Similarly, Leal et al. (38) found a 28.9% prevalence of *Hepatozoon* spp. in *Leptodactylus podicipinus* and *Leptodactylus chaquensis* from two regions of the Pantanal, state of Mato Grosso do Sul, Brazil (38). Readel and Goldberg (39) documented a 17% infection rate in frogs screened in Uganda, with infections from *Hepatozoon*, *Trypanosoma*, or microfilariae. In addition, Al-Khamesi et al. (40) observed a 13.33% prevalence of *Hepatozoon* spp. in 70 adult common frogs collected from different locations in Baghdad, Iraq (40). Netherlands et al. (2) reported a 8.9% prevalence in hyperoliid frogs from northern KwaZulu-Natal, South Africa. In contrast, Parejo-Pulido *et al*. (33) did not detect haemosporidian and haemogregarine parasites in 86 amphibian blood samples from southwestern Iberia. Similarly, Seabra-Babo et al. (56) failed to amplify DNA of haemogregarines in amphibian blood samples from Europe and North Africa, despite clear visual identification in blood smears. The absence of detected parasites in these studies could be attributed to a lack of appropriate aquatic vectors in their study areas or limited exposure to terrestrial vectors [(41)]. These findings highlight the limited information available on the prevalence of *Lankesterella* sp. and *Hepatozoon* spp., especially in Pakistani amphibians and globally. This underscores the importance of exploring these under-researched areas and screening wild hosts from various geo-climatic regions to enhance our understanding of their pathogenicity and diversity, which could inform effective control measures in amphibians.

The primers used in this study were originally designed by Ujvari *et al*. (22) to target Hepatozoon parasites. However, these primers are non-specific and also amplify other genera such as *Eimeria, Sarcocystis* and *Isospora*, as well as various other genetic groups (35). Out of 17 amplified PCR products, only 6 were successfully sequenced, with the remaining products failing due to insufficient blood volume or poor quantity or quality of extracted DNA. Of the six successfully sequenced samples, one *Hepatozoon* spp. was identified from a frog (*H. tigerinus*), while two *Lankesterella* spp. was identified from *H. tigerinus* and one from the toad *B. stomaticus.* Consequently, phylogenetic analyses were performed separately for the two detected parasite genera.

No prior attempts have been made to assess the genetic diversity of *Hepatozoon* spp. among Pakistani anurans. Thus, the 18S rRNA gene sequences from the amplified PCR products were utilized for phylogenetic analysis. The 18S rRNA gene is commonly used in molecular studies to reconstruct the evolutionary histories due to its slow rate of evolution, making it suitable for tracing ancient divergences (23). The DNA sequence analysis indicated that the *Hepatozoon* species infecting Pakistani anuran was 99.77% similar to *H. ixox*o. The haplotype (GenBank aaccession number PP481405) obtained in this study showed similarity to 18S rRNA sequences of *Hepatozoon* spp. isolated from amphibians and reptiles from South Africa by Cook et al. (42) (GenBank accession numbers MG519501 and MG519504), Netherlands et al. (32), (GenBank accession numbers KP119772 and KP119773), and Netherlands et al. (2) (GenBank accession numbers MG041591, MG041598, and MG041600), adeleorinid coccidians in Canada by Barta et al. (43) (GenBank accession numbers JN181157, HQ224960, and HQ224962), snakes in Brazil by Úngari et al. (36) (GenBank accession number MW591556), and bullfrogs in the United States by Mathew et al. (44) (GenBank accession number AF176837) (Figure 2).

Three amplified PCR products from the 18S rRNA gene were used for the phylogenetic analysis of *Lankesterella* spp. The haplotypes identified in this study resided in distinct clades indicating sequence variations within the parasite (Figure 3). *Lankesterella* lineage PP476386 was most similar to sequences isolated from bird species unlike the other lineage (PP544154 and PP541577). Specifically, the Pakistani haplotypes PP544154 and PP541577 clustered with *Lankesterella* sp. reported in lizards in Spain [GenBank accession number KJ131417 (45)] and reptiles in Yemen [GenBank accession number MW076442 (46)]. In contrast, the Pakistani isolate PP476386 clustered with *Lankesterella* sp. from reptiles in Oman [GenBank accession number KX453649 (47)] (Figure 3). This data suggests that while the Pakistani haplotypes exhibit distinct phylogenetic relationships, they are closely related to known *Lankesterella* species found in various reptiles. This may indicate that the local haplotypes could represent new taxonomic entities, reflecting differences in host–parasite compatibility and potentially revealing undescribed endemic species. Our results underscore the influence of host ecology and relatedness on *Lankesterella* species distributions. More broadly, they highlight the necessity of screening wild hosts from remote and underexplored regions of Pakistan to gain deeper insights into parasite diversity.

Both *Hepatozoon* spp. and *Lankesterella* spp. belong to the order Eucoccidiorida (subclass Coccidia, class Conoidasida, Apicomplexa) and collectively considered as Haemogregarines (48). We have mentioned above that our primers were generalized and they are capable of amplifying both *Hepatozoon* spp. and *Lankesterella* spp. and as we were not successful in DNA sequencing all the amplified partial 18S rRNA gene sequences that were amplified in this study, so we were unable to analyze the data separately for *Hepatozoon* spp. and *Lankesterella* spp. Hence, we have analyzed all of our data for Haemogregarines prevalence. In this study, Haemogregarines infection among Pakistani anurans did not vary significantly between different sampling sites (Table 1). This finding aligns with the results of Leal *et al*. (38) who reported similar non-significant variations in *Hepatozoon* sp. prevalence among *Leptodactylus podicipinus* and *Leptodactylus chaquensis* frog species across different sampling sites. However, our results contrast with Readel and Goldberg (39), who observed significant differences in *Hepatozoon* spp. prevalence among different frog species in Uganda. This disparity may be attributed to varying habitat preferences and associated differences in vector abundance and contact rates. *Hepatozoon* spp. are typically transmitted through the ingestion of infected mosquitoes, and frogs living in more aquatic environments might experience higher blood parasite burdens due to increased exposure to insect vectors (39). Regarding the association between parasite infection and the sex of the amphibians, our study found no significant difference in *Hepatozoon* spp. prevalence between males and females across the anuran species (*H. tigerinus*, *B. stomaticus*, *B. olivaceous*, and *B. melanostictus*) captured from different districts (Table 4). This contrasts with the findings of Mohamed and Osman (49), who reported higher *Hepatozoon* sp. prevalence in males compared to females of *A. regularis* toads. The reasons for the observed differences in infection rates between sexes in their study remain unclear, and the authors recommended further screening of toad samples to validate these findings.

Among protozoan diseases, toxoplasmosis is particularly significant due to its zoonotic nature, with *T. gondii*, the causative agent, known to infect nearly all warm-blooded animals (16). Although *T. gondii* is not typically associated with cold-blooded animals, its presence has been documented in marine mammals such as cetaceans, pinnipeds, sirenians, and sea otters (50–52). This raises concerns about the potential role of cold-blooded animals, such as frogs, toads, turtles, crocodiles, snakes, fish, and shellfish, as reservoirs for *T. gondii* (53).

Our study reports a 4.5% prevalence of *T. gondii*-like infections in Pakistani anurans for the first time, detecting the parasite in both frog (*H. tigerinus*) and toad (*B. stomaticus*) species (Table 3). These findings highlight the importance of advanced diagnostic techniques, such as PCR, in understanding the prevalence and transmission dynamics of *T. gondii* and genetically related strains among frog and toad populations, with implications for both animal and public health.

This study also represents the first report on the genetic diversity of *T. gondii*-like strains among Pakistani amphibians. Phylogenetic analysis was performed using PCR-amplified products from the *ITS-1* partial sequence of the pathogen, a commonly targeted marker in molecular phylogenetic studies due to its ease of amplification and relatively high variability (54). The identified haplotypes clustered together, forming a distinct clade separate from all published *T. gondii* sequences. The isolates included in this analysis originated from a variety of hosts across different geographical regions, underscoring the widespread distribution of *T. gondii*-like strains and their potential transmission among diverse host species.

Importantly, while the strains identified in this study are genetically related to *T. gondii*, they do not belong to the reference pathogenic strain, and their pathogenicity remains uncertain. However, these strains found in aquatic animals may represent ancestral species of those currently present in terrestrial animals, which could pose a significant threat due to their pathogenic potential. This highlights the need for further research to evaluate the health risks associated with these genetically related strains. Our findings illustrate the evolution of this parasite across various hosts, shedding light on its adaptability and potential reservoirs within amphibian populations. Understanding the evolution and diversity of *T. gondii*-like strains is essential for informing future control measures and public health strategies.

Our risk factor analysis indicated that the prevalence of *T. gondii*-like strains varied between sampling sites and among different anuran species. However, the prevalence was not associated with the sex of the amphibians screened (Tables 4). As this is the first report regarding the presence of *T. gondii*-like strains among amphibians, direct comparisons with previous studies are not possible. Nonetheless, these findings pave the way for further research into this widely distributed parasite across diverse amphibian species globally. Expanding this research will enhance our understanding of *T. gondii* and related strains in various animal hosts and elucidate host–parasite interactions, potentially leading to more effective control measures for this common pathogen. We recommend employing serological assays for the detection of *T. gondii*-like strains in frog and toad blood and suggest future studies attempt to isolate these strains from the muscles and brain of anurans for definitive confirmation.

## 5 Conclusion

This study provides the first documented evidence of *T. gondii*-like strains, *Hepatozoon* sp., and *Lankesterella* sp. infections in amphibians from Pakistan, revealing significant insights into parasite diversity and prevalence. The detection of *T. gondii*-like strains in both frogs and toads, along with the identification of *H. ixoxo* and *Lankesterella* spp., highlights the complex interactions between amphibians and their parasitic pathogens. Molecular analyses indicated notable genetic diversity among these parasites, suggesting the presence of potential new taxonomic entities and underscoring the necessity for further research. The variation in parasite prevalence across different sampling sites and amphibian species, combined with the lack of sex-specific infection patterns, reflects the dynamics of parasite transmission within amphibian populations. Importantly, the genetically related strains identified in aquatic animals may represent ancestral species of those currently found in terrestrial animals, posing a significant threat due to their pathogenic potential. Our findings contribute to a broader understanding of the evolution of *T. gondii*-like strains and *Hepatozoon* spp. in wildlife, emphasizing the potential implications of our studied hosts in this evolutionary context. This study enhances awareness of animal health and potential zoonotic risks, highlighting the importance of amphibians as reservoirs for these parasites. Future research should explore these relationships further to improve management strategies and reduce the impact of these parasites on amphibian health and ecosystems.

## Data Availability

The datasets generated and/or analyzed during the current study are available in the GenBank repository, with accession numbers PP481405 (*Hepatozoon* sp.), PP544154, PP541577, and PP476386 (*Lankesterella* sp.) and OR896860, OR896861, and OR896862 (*T. gondii*-like strains).

## Footnotes

^1^Biomatters, www.geneious.com
